# 215. *Candida glabrata* (*CG*) Bloodstream Infections (BSIs) Are Characterized by Genetically Diverse Populations of Strain Variants Recovered from Positive Blood Cultures (BCs)

**DOI:** 10.1093/ofid/ofab466.417

**Published:** 2021-12-04

**Authors:** Badrane Hassan, Shaoji Cheng, Guojun Liu, Cornelius J Clancy, Minh-Hong Nguyen

**Affiliations:** University of Pittsburgh, Pittsburgh, Pennsylvania

## Abstract

**Background:**

The long-standing paradigm is that almost all BSIs stem from a single, clonal organism (“single-organism” hypothesis). We hypothesized that *CG*-positive BCs were comprised of genetically diverse strain variants.

**Methods:**

Five to ten *CG* colonies were isolated from positive BC bottles from ten distinct patients (pts) for a total of 94 clones, which underwent NextGen short-read sequencing (Illumina). Variants were analyzed using SNPeff, and a phylogeny was constructed with Maximum Likelihood method.

**Results:**

BCs harbored a diverse population of *CG* strains that were unique to each pt [Fig. 1]. All strains were genetically distinct, differing by unique SNPs and insertions-deletions (indels) [Fig. 2-3]. SNPs were ~8-fold more common than indels. Individual genomes from the same time point in the same pt exhibited consistent magnitude of variations relative to reference genome; however, variations were unique, pointing to significant genomic variability that could be both intra- and interclonal. The number of variant sites for within-pt pairwise clone comparisons ranged from 1924-8500. There were 124,145 variant sites when all clones are compared. Roughly half of all SNPs were identical in different samples from a given pt; the remainder were present/absent in at least one sample per pt. Long-read WGS revealed strains with structural variants in each pt, including chromosomal recombinations and gene duplications that were not evident by short-read WGS. A genomic phylogeny construction showed that 94 clones spanned 3 distinct clades that were distinct from the reference strain. Finally, comparison of non-synonymous mutations among intra-pt clones showed overwhelming overrepresentation of adhesin and adhesin-like genes, pointing to possible importance in host adaptation.

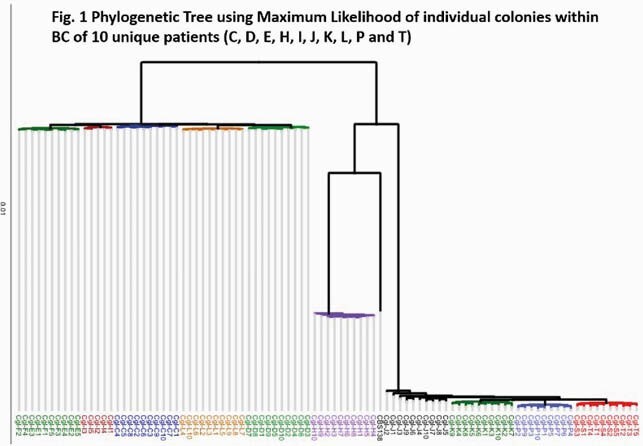

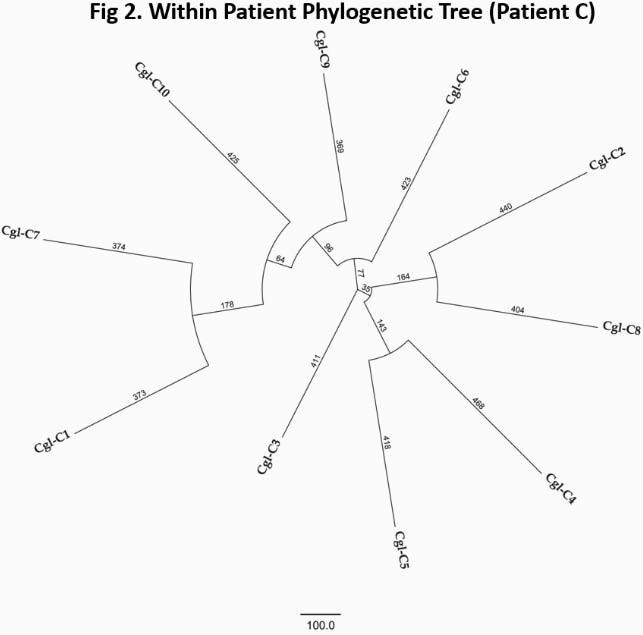

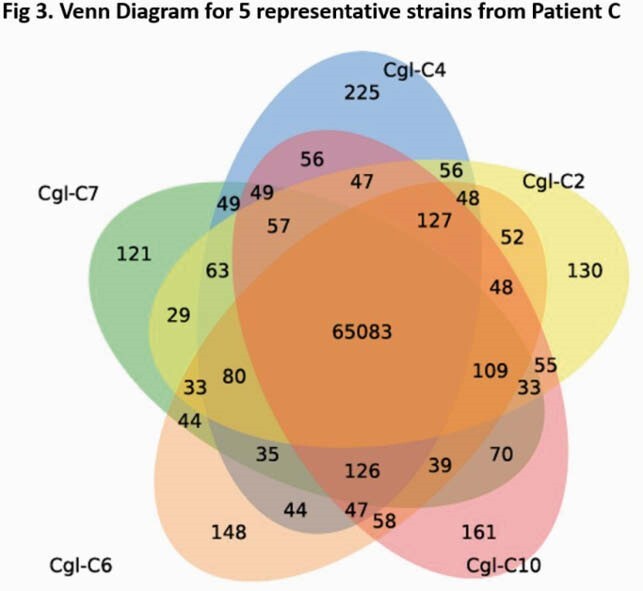

Venn diagram of core genome SNPs of 5 Candida glabrata recovered from blood cultures of patient C.

**Conclusion:**

Preliminary analyses revealed genetically diverse populations of *C. glabrata* in BCs of individuals with BSIs. We are studying phenotypic diversity of strain variants, and the clinical implications of genetically diverse *Candida* BSIs.

**Disclosures:**

**Cornelius J. Clancy, MD**, **Merck** (Grant/Research Support) **Minh-Hong Nguyen, MD**, **Merck** (Grant/Research Support)

